# Supplementary material for: Preparation of Polyaniline/Emulsion Microsphere Composite for Efficient Adsorption of Organic Dyes
Source: Polymers (Basel). 2020 Jan 9;12(1):167. doi: 10.3390/polym12010167 (PMC7023397; doi:10.3390/polym12010167)
Supplement: Supplementary file 1 [file polymers-12-00167-s001.zip › polymers-645187-supplementary.pdf]

Supporting Information

Preparation of Polyaniline/Emulsion Microspheres Composite for Efficient Adsorption of Organic Dyes

Yuanli Liu, Liushuo Song, Linlin Du, Peng Gao, Nuo Liang, Si Wu, Tsuyoshi Minami, Limin Zang, Chuanbai Yu* and Xu Xu*

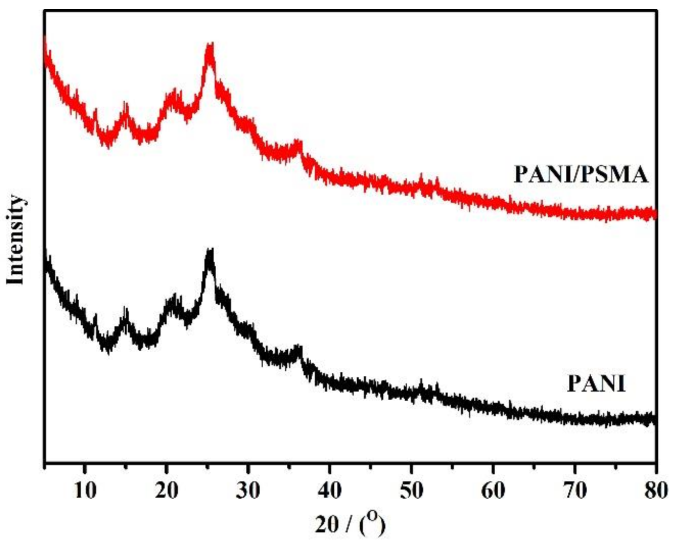

Figure S1. The XRD pattern of PANI and PANI/PSMA.

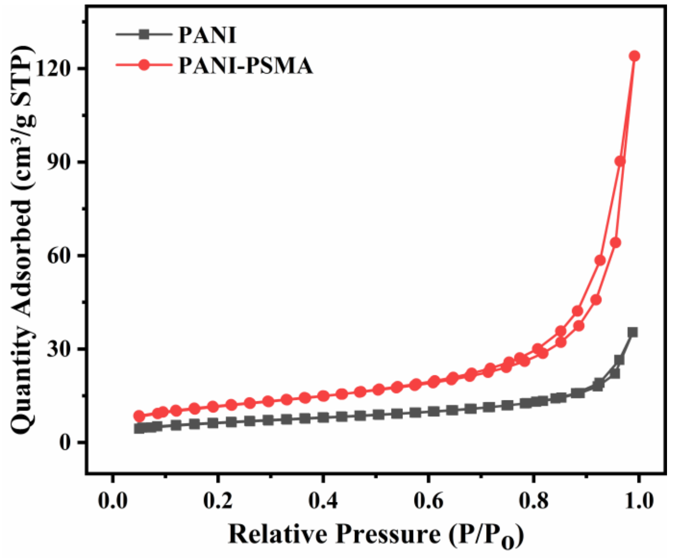

Figure S2. The N_2 adsorption-desorption curves of PANI and PANI/PSMA.

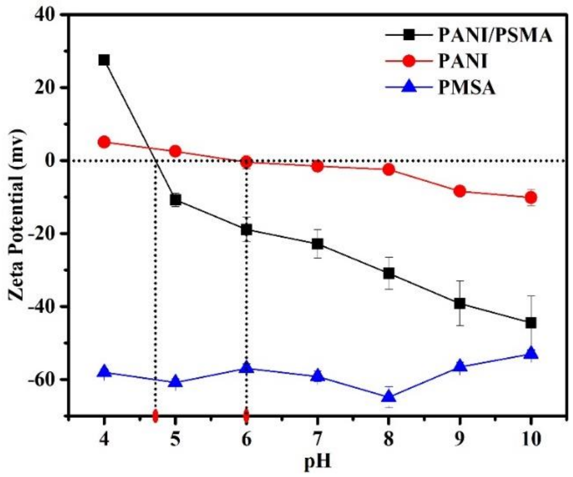

Figure S3. The Zeta potential curves of PANI, PSMA and PANI/PSMA.

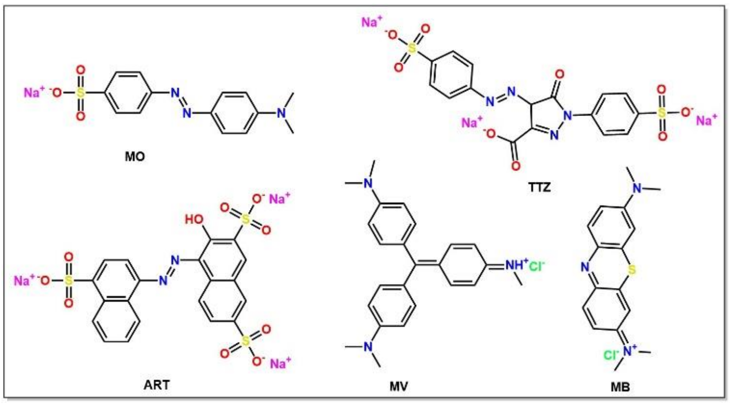

Figure S4. The structure of anionic dyes (MO, TTZ, ART) and cationic dyes (MB, MV).

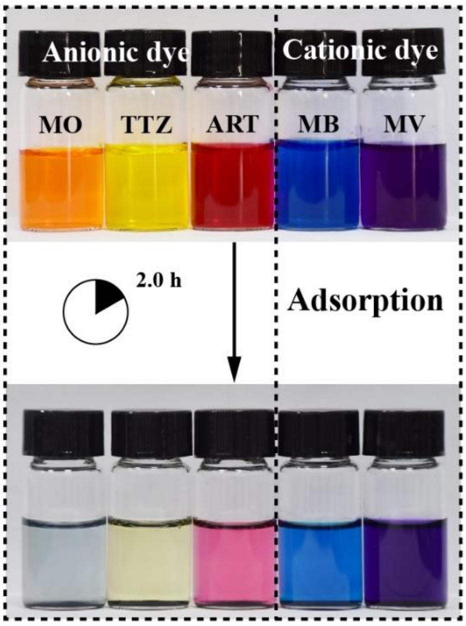

Figure S5. The images of different dyes before and after adsorption by PANI/PSMA.

Table S1. Comparison of adsorption capacity of PANI/PSMA with other adsorbents.

Adsorbents	Dye types	Q_{max} (mg/g)	References
**PANI-PSMA**	MO	**147.93**	**Present study**
Immobilized PANI	MO	77.5	[1]
Suspended PANI	MO	125	[1]
Polyaniline nanofibers	MO	25	[2]
PANI-HNTs	MO	87.03	[3]
Polyaniline nanotubes base/silica composite	MB	10.31	[4]
PANI / PA 6 composite fiber	MO	58.7	[5]
biochar adsorbent	MO	39.37	[6]
amorphous carbon nanotubes	MO	21.51	[7]
CuO/NaA zeolite	MO	79.49	[8]
$CoFe_2O_4$/LDH nanocomposites	MO	137	[9]
graphene oxide	MO	16.83	[10]
furfural residue	MO	54.95	[11]
goethite impregnated with chitosan beads	MO	84	[12]
CO_3O_4 nanoparticles	MO	46.08	[13]
LDH@Fe_3O_4/PVA NC 6 wt%	MO	19.59	[14]
UiO-66-NH_2	MO	86.2	[15]
Modified silkworm exuviae	MO	87.03	[16]
MWCNTs	MO	51.74	[17]
Magnetic Maghemite/Chitosan Nanocomposite Films	MO	28.94	[18]
Porous Carbon	MO	337.8	[19]
N6-PANI nanocomposite web	MO	370	[20]

Table S1 listed the adsorption capacity of PANI-PSMA and other adsorbents. It was shown that the adsorption capacity of PANI-PSMA is higher than that of most other types of adsorbents[1-18].Although some absorption systems with even higher adsorption capacity have been developed [19,20],those absorbents, however, either suffer from a harsh manufacturing process, such as high temperature[19],or involving in heavily use of organic solvents and strong acids[20],which generate substantial quantities of waste liquids and results in adverse effects on the environment, the drawbacks inevitably hindered the large-scale production and application. In contrast, the preparing process of PANI-PSMA does not involve in harsh conditions or use any organic solvents or acids, therefore, the as-prepared PANI-PSMA adsorbent provides a good alternative for effective removal of organic dyes from aqueous solution.

References

1. Bahrudin, N.N.; Nawi, M.A.; Ismail, W.I.N.W. Physical and adsorptive characterizations of immobilized polyaniline for the removal of methyl orange dye. *Korean J. Chem. Eng.* **2018**, *35*, 1450-1461.
2. Duhan, M.; Kaur, R. Adsorptive removal of methyl orange with polyaniline nanofibers: An unconventional adsorbent for water treatment. *Environ. Technol.* **2019**, 1-14.
3. Amer, W.A.; Omran, Mohamed M.; Rehab, A.F.; Ayad, M.M. Acid green crystal-based in situ synthesis of polyaniline hollow nanotubes for the adsorption of anionic and cationic dyes. *RSC Adv.* **2018**, 8, 22536-22545.
4. Ayad, M.M.; Abu El-Nasr, A.; Stejskal, J. Kinetics and isotherm studies of methylene blue adsorption onto polyaniline nanotubes base/silica composite. *J. Ind. Eng. Chem.* **2012**, *18*, 1964-1969.
5. Xia, Y.; Li, T.; Chen, J.; Cai, C. Polyaniline (skin)/polyamide 6 (core) composite fiber: Preparation, characterization and application as a dye adsorbent. *Synth. Met.* **2013**, *175*, 163-169.
6. Yu, J.; Zhang, X.; Wang, D.; Li, P. Adsorption of methyl orange dye onto biochar adsorbent prepared from chicken manure. *Water Sci. Technol.* **2018**, *77*, 1303-1312.
7. Banerjee, D.; Bhowmick, P.; Pahari, D.; Santra, S.; Sarkar, S.; Das, B.; Chattopadhyay, K. Pseudo first ordered adsorption of noxious textile dyes by low-temperature synthesized amorphous carbon nanotubes. *Phys. E* **2017**, *87*, 68-76.
8. Mekatel, E.H.; Amokrane, S.; Aid, A.; Nibou, D.; Trari, M. Adsorption of methyl orange on nanoparticles of a synthetic zeolite naa/cuo. *C. R. Chim.* **2015**, *18*, 336-344.
9. Palza, H.; Delgado, K.; Govan, J. Novel magnetic $CoFe_2O_4$ / layered double hydroxide nanocomposites for recoverable anionic adsorbents for water treatment. *Appl. Clay Sci.* **2019**, 183, 105350.
10. Robati, D.; Mirza, B.; Rajabi, M.; Moradi, O.; Tyagi, I.; Agarwal, S.; Gupta, V. Removal of hazardous dyes-br 12 and methyl orange using graphene oxide as an adsorbent from aqueous phase. *Chem. Eng. J.* **2016**, *284*, 687-697.
11. Chen, X.; Li, H.; Liu, W.; Zhang, X.; Wu, Z.; Bi, S.; Zhang, W.; Zhan, H. Effective removal of methyl orange and rhodamine b from aqueous solution using furfural industrial processing waste: Furfural residue as an eco-friendly biosorbent. *Colloids Surf., A* **2019**, 583, 123976.
12. Munagapati, V.S.; Yarramuthi, V.; Kim, D.-S. Methyl orange removal from aqueous solution using goethite, chitosan beads and goethite impregnated with chitosan beads. *J. Mol. Liq.* **2017**, 240, 329-339.
13. Uddin, M.K.; Baig, U. Synthesis of co3o4 nanoparticles and their performance towards methyl orange dye removal: Characterisation, adsorption and response surface methodology. *J. Cleaner Prod.* **2019**, *211*, 1141-1153.
14. Mallakpour, S.; Hatami, M. An effective, low-cost and recyclable bio-adsorbent having amino acid intercalated ldh@ fe3o4/pva magnetic nanocomposites for removal of methyl orange from aqueous solution. *Appl. Clay Sci.* **2019**, *174*, 127-137.
15. Lv, S.-W.; Liu, J.-M.; Ma, H.; Wang, Z.-H.; Li, C.-Y.; Zhao, N.; Wang, S. Simultaneous adsorption of methyl orange and methylene blue from aqueous solution using amino functionalized zr-based mofs. *Microporous Mesoporous Mater.* **2019**, *282*, 179-187.

16. Chen, H.; Zhao, J.; Wu, J.; Dai, G. Isotherm, thermodynamic, kinetics and adsorption mechanism studies of methyl orange by surfactant modified silkworm exuviae. *J.Hazard. Mater.* **2011**, *192*, 246-254.
17. Yao, Y.; Bing, H.; Feifei, X.; Xiaofeng, C. Equilibrium and kinetic studies of methyl orange adsorption on multiwalled carbon nanotubes. *Chem. Eng. J.* **2011**, *170*, 82-89.
18. Jiang, R.; Fu, Y.-Q.; Zhu, H.-Y.; Yao, J.; Xiao, L. Removal of methyl orange from aqueous solutions by magnetic maghemite/chitosan nanocomposite films: Adsorption kinetics and equilibrium. *J. Appl. Polym. Sci.* **2012**, *125*, E540-E549.
19. Sun, B.; Yuan, Y.; Li, H.; Li, X.; Zhang, C.; Guo, F.; Liu, X.; Wang, K.; Zhao, X. Waste-cellulose-derived porous carbon adsorbents for methyl orange removal. *Chem. Eng. J.* **2019**, 371, 55-63.
20. Zarrini, K.; Rahimi, A.A.; Alihosseini, F.; Fashandi, H. Highly efficient dye adsorbent based on polyaniline-coated nylon-6 nanofibers. *J. Cleaner Prod.* **2017**, *142*, 3645-3654.
